# Lichen-Derived Bioactive Compounds in Diabetes and Cardiovascular Risk: Experimental Evidence, Mechanisms, and Translational Challenges

**DOI:** 10.3390/biomedicines14092083

**Published:** 2026-09-16

**Authors:** Alfredo Torres-Benítez, Carlo Acierno, Davide Nilo, Roberta Cutellè, Karla Troncoso-Monsalve, Sebastián Aburto-Urriaga, Francisco Novoa-Sánchez, Ferdinando Carlo Sasso, Vincenzo Russo, Alfredo Caturano

**Affiliations:** 1 Carrera de Química y Farmacia, Facultad de Ciencias, Universidad San Sebastián, General Lagos 1163, Valdivia 5090000, Chile; alfredo.torres@uss.cl (A.T.-B.); ktroncosom1@correo.uss.cl (K.T.-M.); saburtou@correo.uss.cl (S.A.-U.); fnovoas1@correo.uss.cl (F.N.-S.); 2 Azienda Ospedaliera Regionale San Carlo, 85100 Potenza, Italy; carlo894@gmail.com; 3 Department of Advanced Medical and Surgical Sciences, University of Campania Luigi Vanvitelli, 80138 Naples, Italy; nilodavide@gmail.com (D.N.); ferdinandocarlo.sasso@unicampania.it (F.C.S.); 4 Department of Medical and Surgical Sciences, Magna Græcia University, 88100 Catanzaro, Italy; 5 Division of Cardiology, Department of Medical Translational Sciences, University of Campania Luigi Vanvitelli, 80138 Naples, Italy; 6 Department of Human Sciences and Promotion of the Quality of Life, San Raffaele Roma University, 00166 Rome, Italy

**Keywords:** lichens, secondary metabolites, diabetes, cardiovascular disease, oxidative stress, inflammation, usnic acid

## Abstract

Cardiometabolic disorders arise from interconnected metabolic, inflammatory, oxidative, mitochondrial, and vascular abnormalities. Lichens produce structurally diverse secondary metabolites, including depsides, depsidones, dibenzofurans, anthraquinones, and other phenolic compounds, whose experimental activities intersect several of these pathways. This narrative review examines the chemical diversity, biological actions, and translational potential of lichen-derived compounds in diabetes and cardiovascular risk modulation. Available studies describe antioxidant, anti-inflammatory, enzyme-inhibitory, antiglycation, cytoprotective, and vasomodulatory effects, but the evidence is predominantly based on chemical assays, molecular docking, in vitro experiments, and heterogeneous preclinical models. Direct demonstrations of improved insulin sensitivity, pancreatic β-cell function, endothelial function, lipid handling, or atherosclerosis remain limited, and human efficacy data are lacking. Translation is further constrained by incomplete chemical standardisation, uncertain bioavailability and target-tissue exposure, limited interaction data, and safety concerns. Usnic acid illustrates the central challenge: broad biological activity coexists with mitochondrial toxicity and clinically relevant hepatotoxic potential. Lichen metabolites therefore currently represent pharmacological probes and medicinal-chemistry scaffolds rather than clinically applicable interventions for diabetes or cardiovascular risk reduction. Future development requires compound-specific target validation, reproducible preparations, pharmacokinetic characterisation, functional disease endpoints, and rigorous safety assessment.

## 1. Introduction

Cardiometabolic disorders, including obesity, type 2 diabetes mellitus, metabolic dysfunction-associated steatotic liver disease, dyslipidaemia, hypertension, and atherosclerotic cardiovascular disease, represent interconnected manifestations of metabolic and vascular dysfunction rather than isolated clinical conditions. Their frequent coexistence progressively increases the risk of diabetes-related complications, cardiovascular events, heart failure, chronic kidney disease, and premature mortality [1,2,3,4]. Although contemporary multifactorial management has substantially improved cardiometabolic outcomes, considerable residual risk persists [1,3]. This burden is partly sustained by mechanisms that are not completely addressed by conventional risk-factor control, including chronic low-grade inflammation, oxidative and endoplasmic reticulum stress, mitochondrial dysfunction, adipose-tissue dysfunction, endothelial injury, and maladaptive immunometabolic crosstalk [5,6,7,8]. These processes are closely interconnected: insulin resistance promotes lipotoxicity and inflammation, oxidative stress disrupts insulin signalling and nitric oxide bioavailability, and vascular inflammation accelerates atherogenesis [7,8,9,10]. Molecules capable of modulating multiple components of this network are therefore of pharmacological interest. However, such pleiotropy must be demonstrated experimentally and should not be inferred solely from chemical antioxidant assays, molecular docking, or broad phenotypic effects. Within this broader spectrum, the currently available evidence on lichen-derived compounds is most directly related to diabetes and cardiovascular risk, whereas direct experimental evidence in other cardiometabolic conditions remains particularly limited.

Natural products remain an important source of biologically active molecules and pharmacological scaffolds [11]. Lichens are complex symbiotic associations between a fungal partner and one or more photosynthetic partners and produce a chemically distinctive repertoire of secondary metabolites, many of which are uncommon or absent in higher plants [12,13]. These include depsides, depsidones, dibenzofurans, pulvinic acid derivatives, anthraquinones, and other phenolic compounds involved in ecological defence, photoprotection, and adaptation to environmental stress. Several lichen-derived metabolites and extracts have demonstrated antioxidant, anti-inflammatory, antimicrobial, enzyme-inhibitory, and cytotoxic activities in experimental systems [12,13,14,15]. Their chemical diversity and potential to influence interconnected biological pathways provide a rationale for investigating their possible relevance to glucose regulation and cardiovascular risk-related mechanisms [16]. Nevertheless, substantial limitations complicate interpretation of the available evidence. Most findings derive from cell-free assays, molecular docking, in vitro experiments, or heterogeneous animal models. Extract composition is frequently incompletely characterised, concentrations used in vitro may not be achievable in vivo, and biological effects observed with crude extracts cannot necessarily be attributed to individual metabolites. Moreover, antioxidant or anti-inflammatory activity does not by itself establish improved insulin sensitivity, β-cell function, endothelial function, or atherosclerosis. Usnic acid, the best-characterised lichen metabolite, illustrates the additional tension between biological activity and safety, because its pharmacological effects coexist with mitochondrial uncoupling and clinically relevant hepatotoxic potential [17,18,19].

This narrative review critically evaluates the chemical diversity, mechanistic evidence, and translational potential of lichen-derived bioactive compounds in diabetes and cardiovascular risk modulation. Unlike broader reviews of lichen pharmacology, it examines whether the reported activities are supported by findings directly relevant to glucose regulation, insulin sensitivity, pancreatic β-cell function, vascular biology, lipid-related processes, and atherosclerosis. Particular attention is given to the distinction between evidence obtained with purified compounds and findings based on crude extracts, chemical assays, in silico analyses, or mechanistic extrapolation. Bioavailability, pharmacologically achievable exposure, formulation, and hepatic and mitochondrial safety are also considered. The ultimate aim is to identify the most credible pharmacological leads while distinguishing them from effects that remain preliminary or hypothesis-generating.

## 2. Literature Search

This narrative review was informed by a literature search conducted in PubMed/MEDLINE, Scopus, and Web of Science. The literature search was last updated on 7 September 2026, with no restrictions on publication date. Search terms included combinations of “lichen”, “lichen-derived compounds”, “lichen secondary metabolites”, and representative metabolites, including “usnic acid”, “atranorin”, “lobaric acid”, “salazinic acid”, and “parietin”, together with terms related to diabetes, cardiovascular risk, and relevant biological processes, including “diabetes”, “insulin resistance”, “glucose metabolism”, “obesity”, “cardiovascular disease”, “atherosclerosis”, “endothelial dysfunction”, “lipid metabolism”, “oxidative stress”, and “inflammation”. Reference lists of relevant original articles and reviews were also examined to identify additional publications. English-language original experimental studies, preclinical investigations, and relevant reviews addressing the chemistry, biological activity, pharmacokinetics, safety, or potential relevance of lichen-derived extracts or purified metabolites to diabetes and cardiovascular risk were considered. Priority was given to studies providing compound-specific evidence and functional outcomes relevant to glucose regulation, metabolic tissues, vascular biology, lipid handling, and atherosclerosis. Chemical assays, molecular docking studies, and non-disease-specific experimental models were also considered when useful for describing the available mechanistic or pharmacological evidence, but were not interpreted as direct evidence of antidiabetic or cardiovascular efficacy. Study selection was based on relevance to the objectives of the review and the contribution of each publication to the critical narrative synthesis. Given the narrative design of the review, no formal systematic-review protocol, risk-of-bias assessment, or quantitative evidence synthesis was undertaken.

## 3. Pathophysiological Context: Diabetes and Cardiovascular Risk

Diabetes and cardiovascular disease share several interconnected metabolic, inflammatory, mitochondrial, and vascular abnormalities. Insulin resistance, adipose-tissue dysfunction, oxidative stress, and chronic inflammation contribute to glucose dysregulation, dyslipidaemia, endothelial dysfunction, and atherosclerosis [20,21,22,23]. This shared pathophysiological background provides a rationale for investigating compounds with potentially pleiotropic activity. However, relevance to diabetes or cardiovascular risk should be supported by functional effects in appropriate experimental models and should not be inferred solely from chemical antioxidant activity, molecular docking, or nonspecific cytotoxicity.

### 3.1. Insulin Resistance and Glucose Dysregulation

Insulin resistance is a central feature of the cardiometabolic continuum, involving impaired insulin signalling in skeletal muscle, liver, and adipose tissue, increased hepatic glucose production, altered lipid partitioning, and progressive loss of metabolic flexibility [7,24,25]. Pathways involving IRS-PI3K-Akt signalling, AMP-activated protein kinase (AMPK), peroxisome proliferator-activated receptors (PPARs), and GLUT4 trafficking are therefore relevant experimental targets [26,27]. Nevertheless, inhibition of carbohydrate-digesting enzymes or activity in cell-free assays should not be considered equivalent to improved insulin sensitivity. Direct evidence requires functional outcomes such as insulin-stimulated glucose uptake, suppression of hepatic glucose production, restoration of insulin signalling, or improvement in glucose homeostasis in validated models.

### 3.2. Inflammation and Immunometabolic Dysfunction

Chronic low-grade inflammation, defined as a persistent and modest activation of inflammatory pathways in the absence of overt acute inflammation, contributes to both metabolic and vascular disease. In adipose tissue, liver, and skeletal muscle, inflammatory signalling interferes with insulin action, whereas within the vascular wall it promotes endothelial activation, leukocyte recruitment, and plaque progression [5,28,29]. NF-κB-dependent cytokine production and NLRP3 inflammasome activation represent relevant mechanistic nodes linking nutrient excess and cellular stress to insulin resistance and vascular injury [30,31]. Accordingly, anti-inflammatory effects of lichen-derived compounds should be evaluated in disease-relevant metabolic or vascular models. Reductions in cytokine production in generic inflammatory systems may support biological plausibility but cannot independently demonstrate improved insulin sensitivity or cardiovascular protection.

### 3.3. Oxidative Stress and Mitochondrial Dysfunction

Oxidative and mitochondrial stress contribute to impaired insulin signalling, β-cell dysfunction, endothelial injury, and diabetic complications [6,9,32]. However, reactive oxygen species also participate in physiological signalling, and their biological effects depend on cellular localization, intensity, and duration [33]. Consequently, radical-scavenging activity measured in chemical assays provides preliminary evidence of redox activity but cannot, by itself, demonstrate antioxidant efficacy in biological or intracellular settings [34]. Evaluation of lichen metabolites should therefore incorporate cellular redox status, mitochondrial respiration, membrane potential, ATP production, and cytotoxicity. This distinction is particularly important for usnic acid, whose reported biological activities coexist with mitochondrial uncoupling and hepatotoxic potential [19,35].

### 3.4. Endothelial Dysfunction and Atherosclerosis

Endothelial dysfunction represents a major interface between metabolic abnormalities and cardiovascular disease [36]. Reduced nitric oxide bioavailability, increased oxidative stress, expression of adhesion molecules, and recruitment of inflammatory cells contribute to the initiation and progression of atherosclerosis [8,37]. Vascular relevance of lichen-derived compounds should therefore be supported by direct endpoints such as eNOS activity, nitric oxide production, VCAM-1 or ICAM-1 expression, leukocyte adhesion, vasoreactivity, macrophage lipid handling, or plaque characteristics. Antioxidant or anti-inflammatory activity observed outside vascular models may justify further investigation, but it should not be interpreted as evidence of anti-atherosclerotic efficacy.

## 4. Lichen-Derived Bioactive Compounds: Chemical and Biological Overview

Lichens are symbiotic systems formed by the association between a mycobiont and one or more photobionts [12]. This organisation supports the production of a chemically distinctive repertoire of secondary metabolites, many of which are synthesised predominantly by the fungal partner [15,38,39,40,41]. Their expression and relative abundance may nevertheless vary with symbiotic interactions, environmental conditions, geographical origin, substrate, and associated microorganisms [14,42,43]. This variability is pharmacologically relevant because differences in source material can affect extract composition, reproducibility, and the attribution of biological effects to individual constituents.

Lichen metabolites derive mainly from the acetate–malonate pathway through polyketide synthases, although the mevalonate and shikimate pathways also contribute [15,38,39,44]. These pathways generate depsides, depsidones, dibenzofurans, anthraquinones, pulvinic acid derivatives, terpenoids, and other structurally diverse metabolites. Environmental pressures, including ultraviolet radiation, recurrent desiccation, and microbial competition, contribute to this chemical diversity [12,45,46,47]. However, an ecological function such as photoprotection or chemical defence should not itself be interpreted as evidence of pharmacological activity in mammalian systems.

Although lichen metabolites have been investigated across several pharmacological fields, the activities most relevant to the present review involve redox and inflammatory regulation, carbohydrate-digesting-enzyme inhibition, protein glycation, metabolic signalling, and vascular reactivity [14,15]. Structural features such as phenolic hydroxylation, ester or ether linkages, conformational constraints, and lipophilicity may influence these activities, as well as solubility, membrane partitioning, metabolic stability, and toxicity. Such relationships must nevertheless be evaluated for individual compounds rather than inferred from chemical class alone.

Pharmacological development is further complicated by the low aqueous solubility and marked lipophilicity of some widely investigated lichen metabolites, particularly usnic acid, together with the compositional variability of crude extracts [19,47]. High-resolution LC–MS/MS and metabolomic approaches can improve compound identification, dereplication, and quantitative chemical characterisation [48,49,50]. Integrated metabolomic and transcriptomic approaches may further connect metabolite accumulation with candidate biosynthetic genes, as recently demonstrated in other natural-product systems [51]. Together with the identification of biosynthetic gene clusters and development of heterologous production systems, these approaches may eventually support more reproducible access to selected lichen metabolites, particularly given the slow growth, limited biomass availability, and cultivation difficulties of many lichens [15,39,50,52].

### 4.1. Major Chemical Classes

Before addressing the main classes of lichen metabolites, it is worth noting that their classification is based not only on structural criteria, but also on biosynthetic origin and biological function. Relatively subtle modifications in substitution patterns, such as methylation, hydroxylation, or intramolecular cyclisation, can influence physicochemical properties and biological activity, highlighting the importance of structure–activity relationship (SAR) studies [38,53,54]. Likewise, the coexistence of multiple metabolite families within the same thallus suggests possible synergistic or modulatory interactions, which may explain the greater complexity of biological responses observed in crude extracts compared with isolated compounds [55,56]. Among these families, depsides, depsidones, dibenzofurans, and other phenolic compounds represent the most relevant classes from a chemical and pharmacological standpoint. The principal structural features, representative compounds, and experimentally reported activities are summarized in Table 1.

#### 4.1.1. Depsides

**Figure 1:**
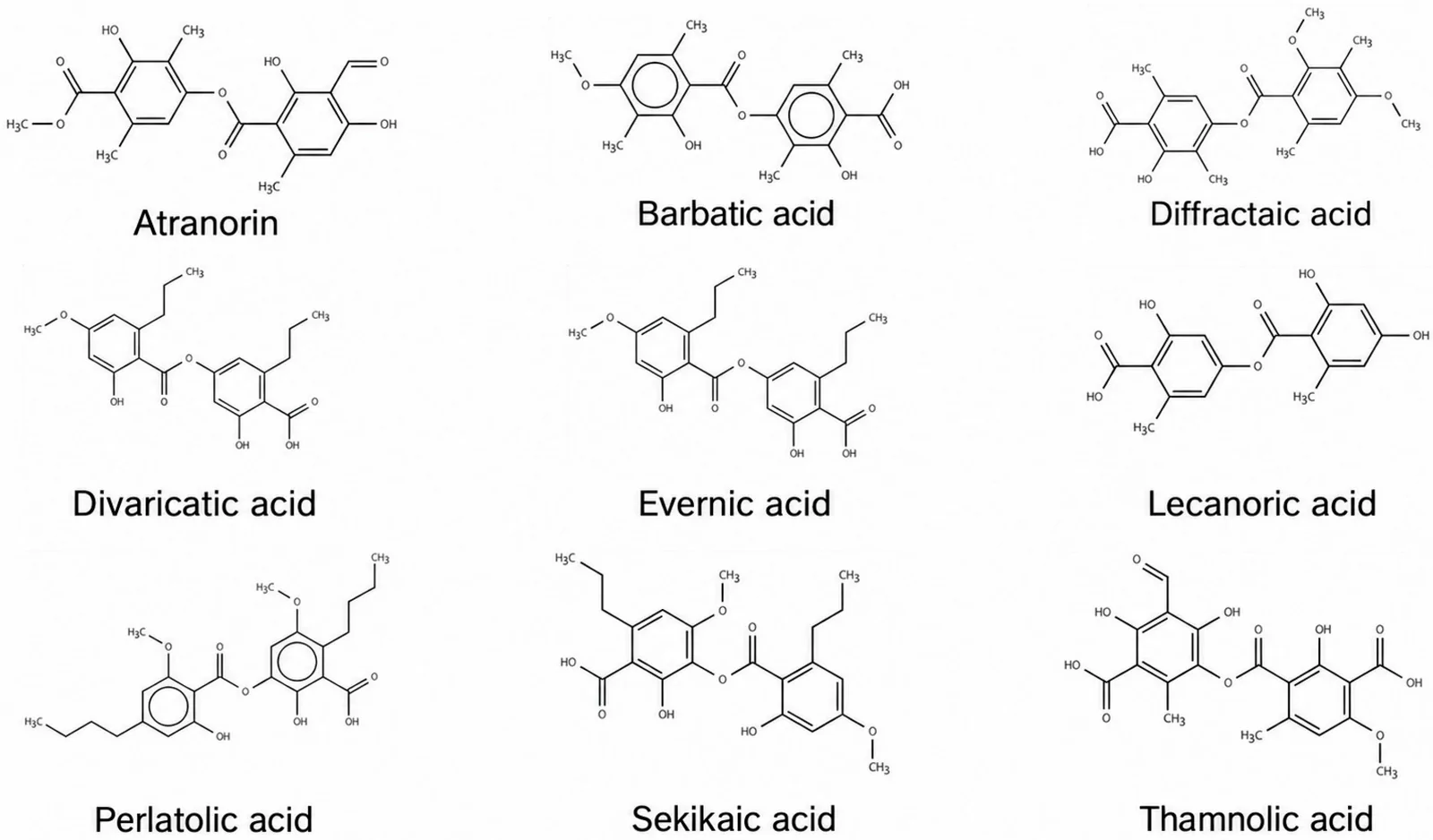
Representative chemical structures of selected lichen depsides. The panel illustrates structural diversity within this ester-linked class, including atranorin, barbatic acid, diffractaic acid, divaricatic acid, evernic acid, lecanoric acid, perlatolic acid, sekikaic acid, and thamnolic acid.

Depsides constitute a key structural and biosynthetic starting point for other related families. They arise from the condensation of two or more phenolic acid units, derived mainly from orsellinic or β-orsellinic acid, linked by ester bonds [38,53,57]. These molecules have a high density of hydroxyl and carboxyl groups, which can contribute to redox activity in cell-free assays [58,59]. Among the most representative examples are atranorin and lecanoric acid, both widely used as chemotaxonomic markers [14,60]. Structurally, depsides show greater conformational flexibility and greater susceptibility to hydrolysis than depsidones, due to the presence of ester bonds [53]. Their phenolic groups can support electron donation and resonance stabilisation, contributing to radical-scavenging and reducing activity in cell-free assays [61,62,63]. However, these chemical properties do not necessarily predict intracellular antioxidant or antimicrobial effects. Beyond these cell-free observations, selected depsides have also been investigated in cellular models of inflammatory and oxidative-stress signalling, including NF-κB- and Nrf2-related pathways [56,64]. Representative structures of major depsides are shown in Figure 1.

#### 4.1.2. Depsidones

**Figure 2:**
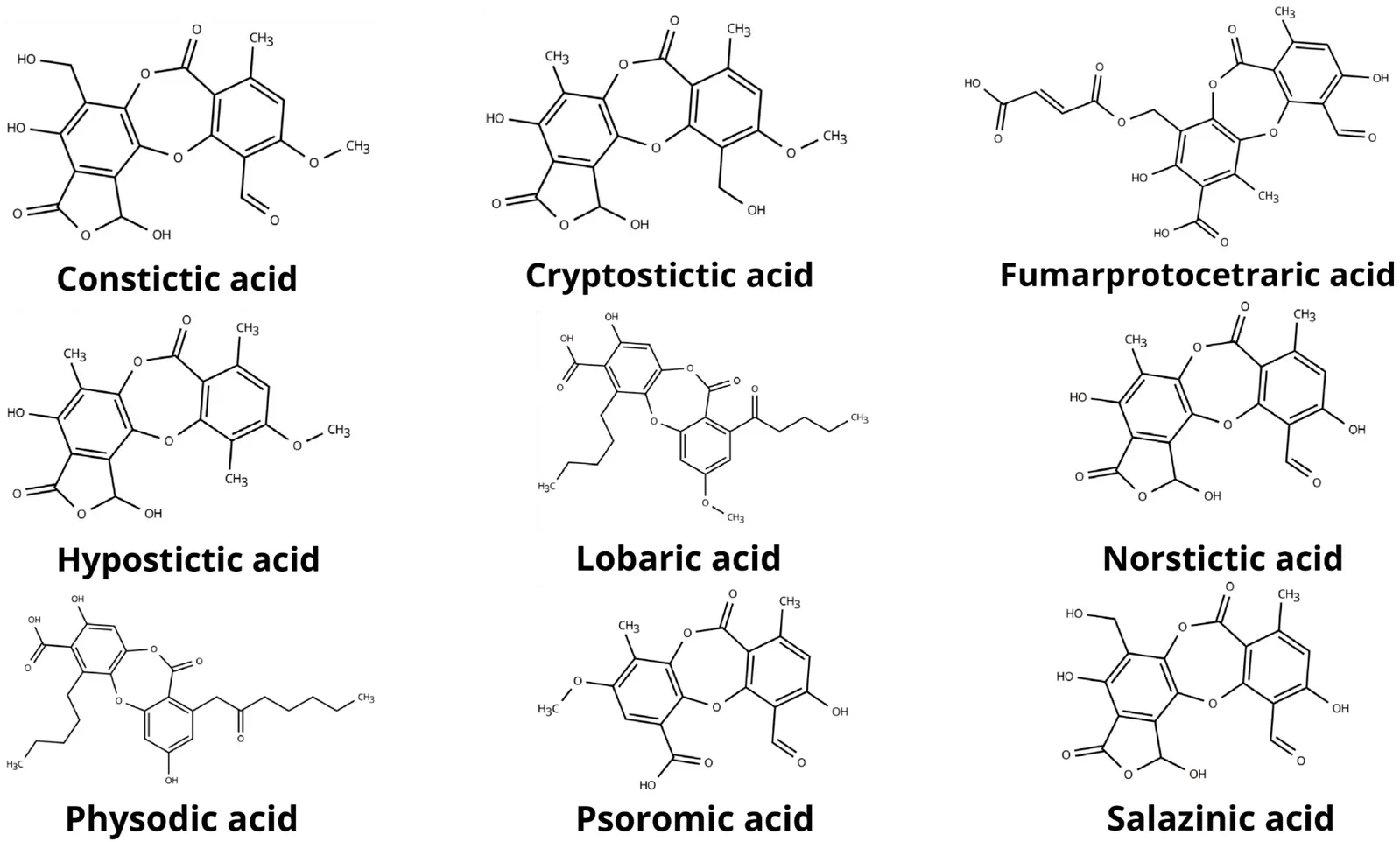
Representative chemical structures of selected lichen depsidones. The examples illustrate the conformationally constrained dibenzodioxepinone-type scaffold and include constictic acid, cryptostictic acid, fumarprotocetraric acid, hypostictic acid, lobaric acid, norstictic acid, physodic acid, psoromic acid, and salazinic acid.

Depsidones are biosynthetically derived from depsides through oxidative cyclisation, often mediated by cytochrome P450 enzymes, generating an intramolecular ether bond that gives rise to a tricyclic dibenzodioxepinone-type system [39,54]. This transformation involves a shift from structures dominated by ester bonds to systems containing ether bonds, which confers greater structural rigidity, chemical stability, and hydrophobic character [40,57]. Representative examples include salazinic, stictic, and fumaroprotocetraric acids, whose structural differences may contribute to variation in their physicochemical and biological properties [54]. Representative structures of major depsidones are shown in Figure 2.

The conformational rigidity of depsidones may influence their interactions with biological targets, but direct comparative structure–activity evidence remains limited. Individual depsidones have shown antimicrobial, antifungal, anti-inflammatory, or enzyme-inhibitory activities in different experimental systems [55,61,65,66]. Salazinic acid, norlobaridone, and other depsidones have also been investigated as inhibitors of lipoxygenases or α-glucosidases [54,66], although their limited solubility and the scarcity of pharmacokinetic data remain obstacles to pharmacological development.

#### 4.1.3. Dibenzofurans

**Figure 3:**
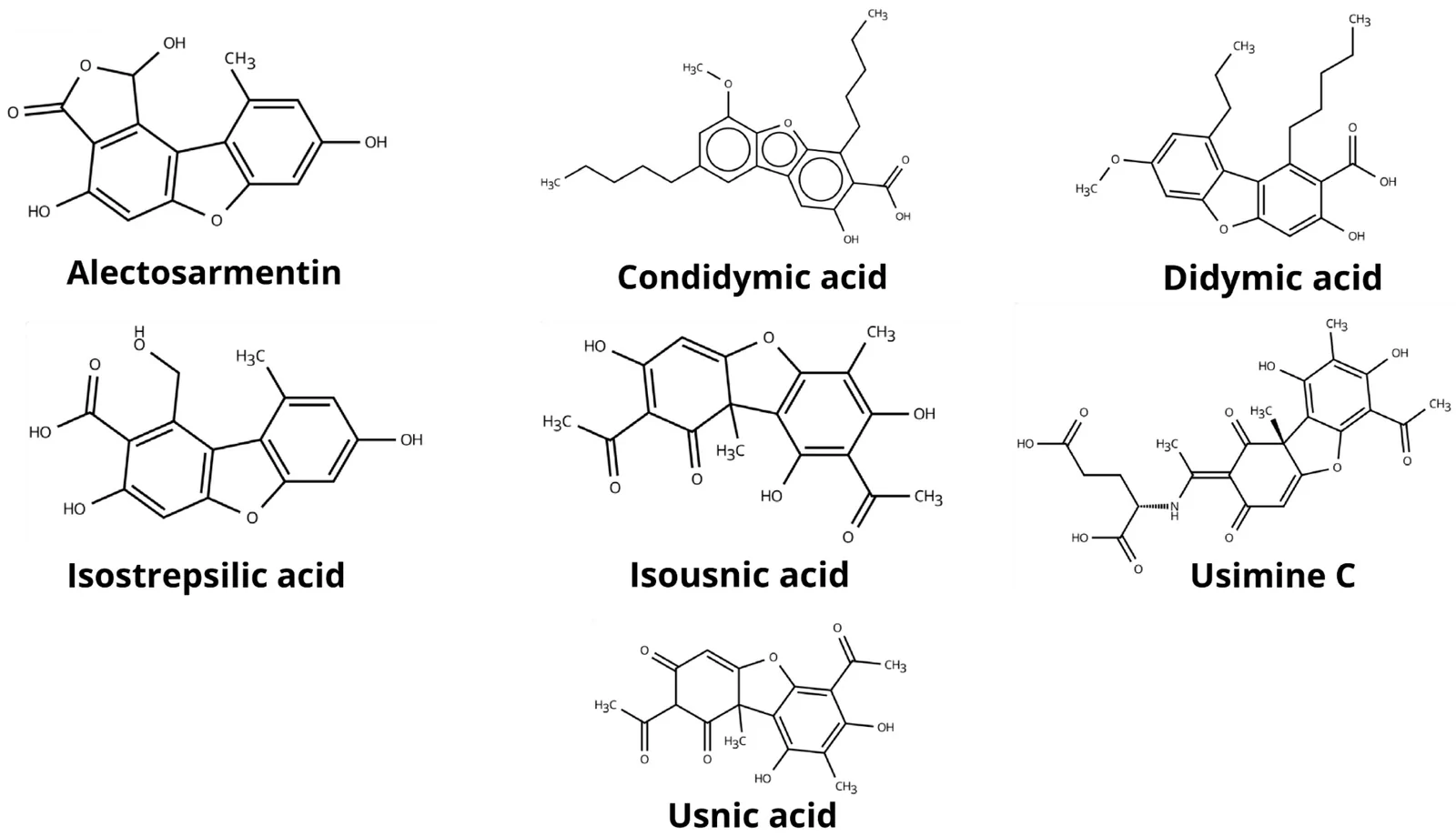
Representative chemical structures of dibenzofurans and structurally related lichen metabolites. Usnic acid is the best-characterised member in pharmacological and toxicological studies; related structures illustrate the chemical diversity surrounding this scaffold.

Dibenzofurans constitute a distinctive structural class derived from the polyketide pathway. Usnic acid is their best-studied representative, both for its broad spectrum of biological activity and for its toxicological profile [14,18,19]. Representative dibenzofurans and structurally related metabolites are shown in Figure 3.

This compound is chiral at position C9b, generating enantiomers with differences in biological activity and toxicity [64]. The marked lipophilicity of usnic acid is a compound-specific physicochemical property that may influence membrane partitioning, aqueous solubility, cellular uptake, tissue distribution, and toxicity. However, these relationships depend on concentration, formulation, biological matrix, and experimental system and should not be interpreted as universal predictors of pharmacological activity [19,40,63]. At sufficiently high exposure, usnic acid acts as an uncoupler of mitochondrial oxidative phosphorylation, dissipating the proton gradient and reducing ATP production, with secondary generation of reactive oxygen species (ROS). Mitochondrial uncoupling and the resulting ATP depletion provide a mechanistic basis for the hepatotoxicity associated with usnic acid and represent a major limitation to its systemic clinical development [19,47,56].

#### 4.1.4. Phenolic Metabolites

**Figure 4:**
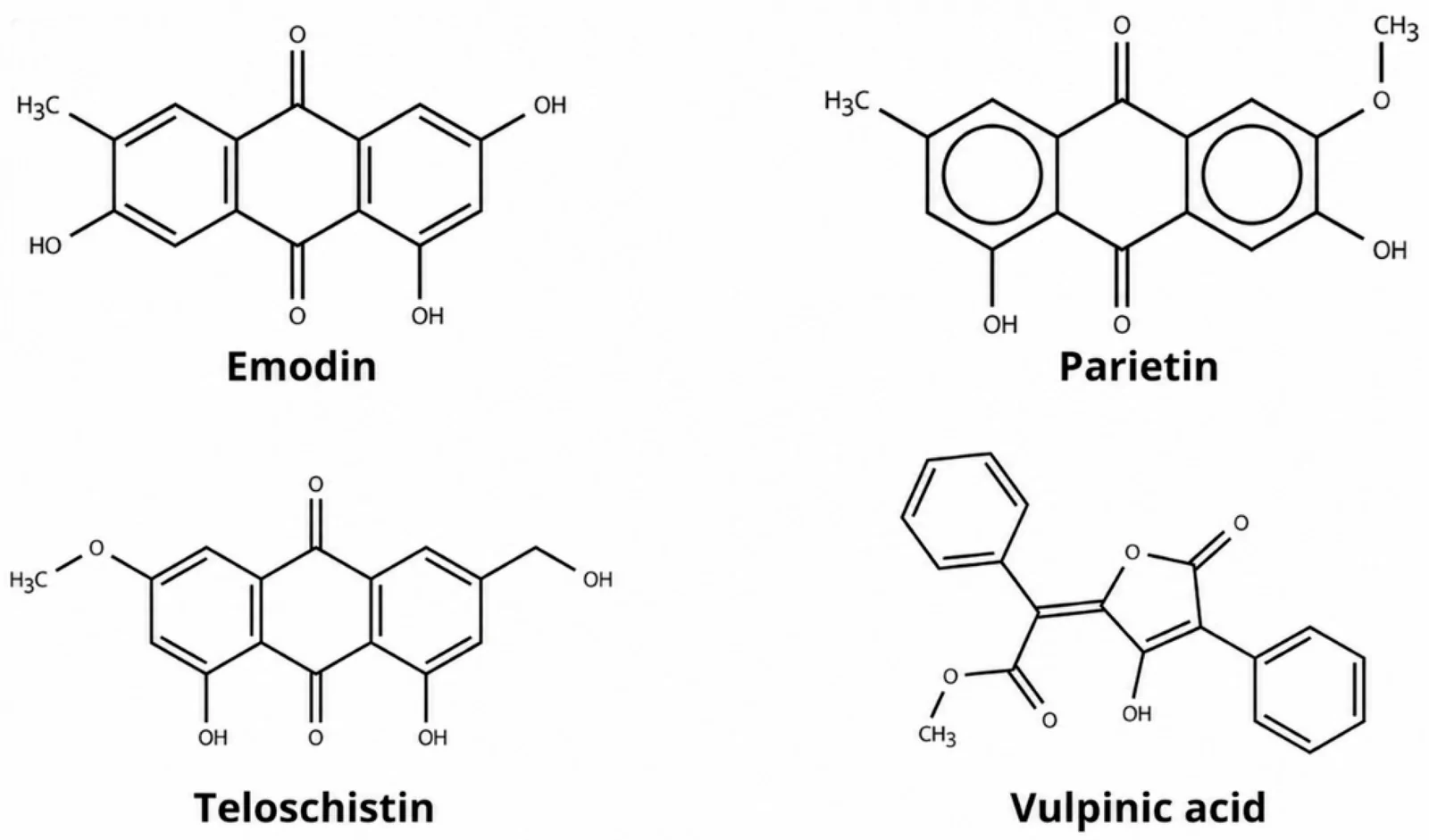
Representative chemical structures of selected anthraquinones, pulvinic acid derivatives, and related phenolic metabolites. Emodin, parietin, teloschistin, and vulpinic acid exemplify highly conjugated molecules associated with photoprotective, redox-active, and other experimentally reported biological properties.

In addition to the classes above, lichens produce a wide variety of phenolic metabolites, including pulvinic acid derivatives, anthraquinones, and other aromatic compounds [14,15,65]. Representative anthraquinones, pulvinic acid derivatives, and related phenolic metabolites are shown in Figure 4.

Among these metabolites, parietin has particular relevance to the present review because it has been examined in a disease-specific animal model reporting integrated glucose- and lipid-related outcomes, as discussed in Section 5.1 [67]. By contrast, the evidence available for vulpinic acid, emodin, teloschistin, and several related metabolites concerns predominantly photoprotective, chemical redox, antimicrobial, or cytotoxic endpoints [12,45,56,59,64]. These findings contribute to chemical and structure–activity characterisation but do not establish effects on glucose homeostasis or cardiovascular risk. The highly conjugated structures of these metabolites remain potentially useful for compound-specific investigation, provided that future studies evaluate functional metabolic or vascular outcomes at achievable and non-toxic exposures.

**Table 1:** Principal chemical classes of lichen-derived metabolites and their potential relevance to diabetes and cardiovascular research.

Class	Structural Features	Representative Compounds	Reported Activities	Main Interpretative Limitation	Refs.
Depsides	Two or more phenolic units linked by ester bonds	Atranorin; lecanoric, evernic, perlatolic, and thamnolic acids	Redox activity; anti-inflammatory effects; enzyme inhibition	Activity is sensitive to substitution patterns; many findings derive from chemical or general cellular assays	[38,53,57,58,59]
Depsidones	Oxidatively cyclized depsides containing ester and ether linkages	Salazinic, stictic, norstictic, lobaric, physodic, and psoromic acids	Anti-inflammatory activity; lipoxygenase and α-glucosidase inhibition	Low aqueous solubility and limited exposure data; diabetes- and cardiovascular-related functional endpoints are scarce	[54,65,68,69]
Dibenzofurans	Fused tricyclic scaffold; usnic acid is chiral at C9b	Usnic acid; isousnic acid; didymic acid	Anti-inflammatory, antimicrobial, redox-active, and cytotoxic effects	Mitochondrial uncoupling and hepatotoxicity narrow the translational window	[14,18,19,63,64]
Other phenolic metabolites	Anthraquinone, pulvinic acid, and related conjugated scaffolds	Emodin; parietin; teloschistin; vulpinic acid	Photoprotection; redox activity; selective cytotoxicity	Evidence is predominantly unrelated to diabetes or cardiovascular disease and often based on broad phenotypic assays	[12,14,15,45,59]

Abbreviations: C9b, carbon atom at position 9b; α-glucosidase, alpha-glucosidase.

### 4.2. Cross-Cutting Biological Activities

Anti-inflammatory and antioxidant activities are among the most consistently reported properties of lichen extracts and isolated metabolites [70]. In cell-based and animal models, selected preparations have reduced neutrophil migration, inflammatory mediator production, and NF-κB- or NLRP3-related signalling, whereas purified compounds such as usnic acid and lobaric acid have shown activity in specific inflammatory systems [69,71,72,73,74]. In parallel, depsides, depsidones, and other phenolic metabolites display radical-scavenging and reducing activity in chemical assays, partly related to the presence and position of free phenolic hydroxyl groups [58,62,75,76]. These findings identify inflammation and redox regulation as cross-cutting areas of pharmacological interest, but their relevance depends strongly on the experimental system and the nature of the tested material.

Interpretation requires a clear distinction between crude extracts, chemically characterised fractions, and purified metabolites. Extract-level effects may reflect additive, synergistic, or antagonistic interactions among multiple constituents, including interactions that modulate toxicity, and therefore cannot be confidently attributed to individual compounds without adequate chemical characterisation and fractionation [77]. Likewise, molecular docking can prioritise candidate interactions but does not demonstrate target engagement, while cell-free antioxidant assays measure chemical reactivity under defined experimental conditions rather than intracellular activity [34,78,79]. Findings obtained in generic inflammatory or non-disease-specific animal models require confirmation in metabolic and vascular systems at pharmacologically relevant, non-cytotoxic exposures [80]. The following sections therefore examine whether these experimental activities translate into functional findings relevant to glucose regulation, metabolic tissues, endothelial function, lipid handling, or atherosclerotic processes.

The evidence examined in the following disease-focused sections derives from heterogeneous experimental settings, including cell-free enzyme assays, in silico analyses, cell-based experiments, non-disease-specific animal studies, disease-specific animal models, and pharmacokinetic or toxicological investigations. These study types address different experimental questions and are therefore described according to what they directly demonstrate. Disease-relevant functional outcomes are distinguished from mechanistic plausibility or hypothesis-generating observations, without applying a formal evidence hierarchy or grading system. Beyond this distinction between experimental settings, translational progression also requires movement from broad phenotypic or computational activity towards compound-specific target engagement and causal pathway validation. Recent natural-product research illustrates the increasing use of experimentally defined molecular pathways to connect bioactive compounds with downstream cellular responses [81]. A comparable level of validation will be required before proposed effects involving AMPK, Nrf2, NF-κB, NLRP3, PI3K–Akt, or eNOS can be confidently assigned to individual lichen metabolites.

**Figure 5:**
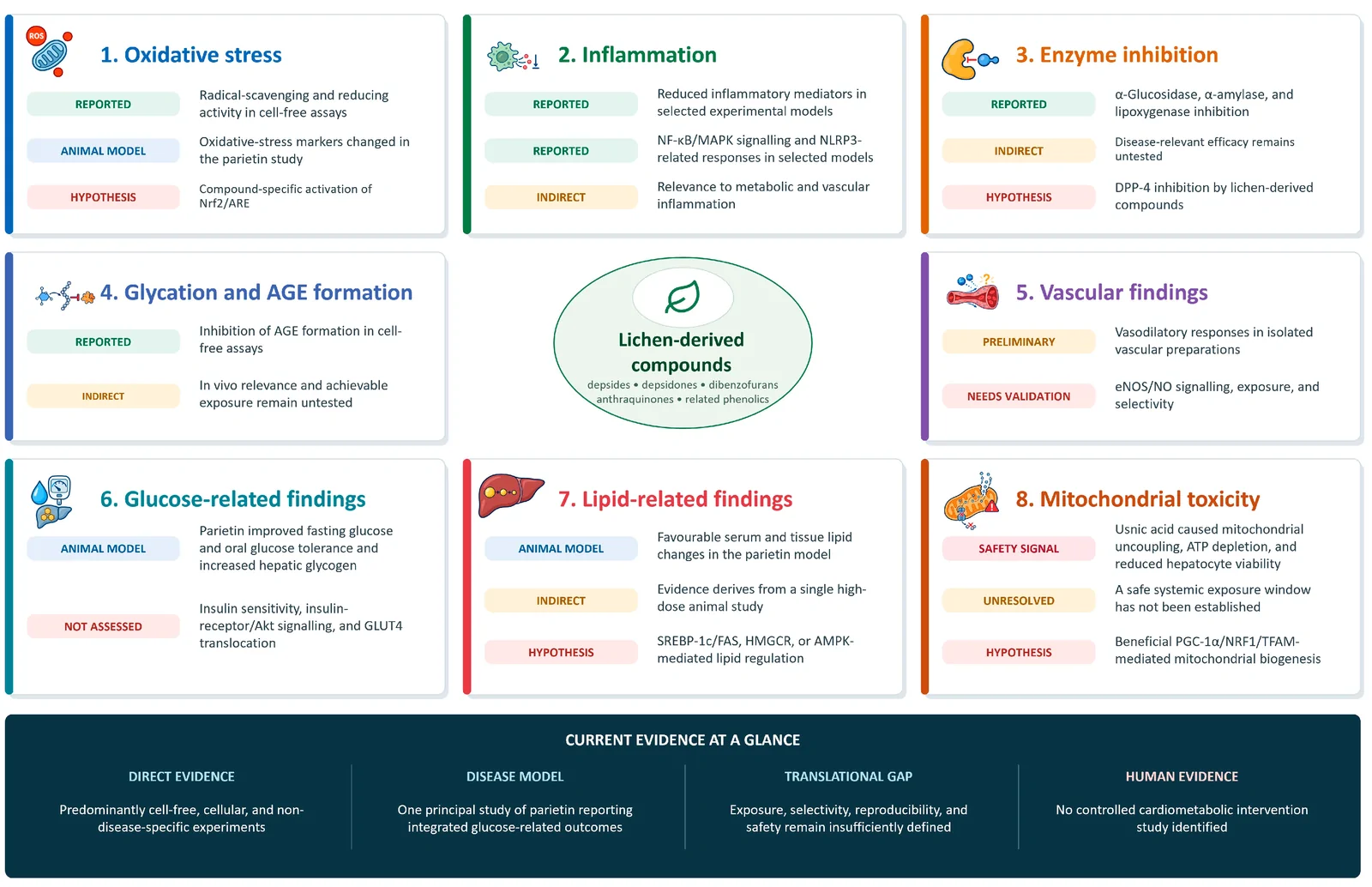
Experimental evidence and translational gaps for lichen-derived compounds in diabetes and cardiovascular risk. The figure summarises the principal biological activities and disease-related findings reported for lichen-derived extracts and metabolites. Evidence labels distinguish directly reported findings, observations from animal models, indirect or preliminary evidence, hypotheses, mechanisms that were not assessed or require further validation, unresolved questions, and safety signals. These descriptors clarify the experimental context and should not be interpreted as a formal evidence-grading hierarchy. Current evidence derives predominantly from cell-free, cellular, and non-disease-specific experimental systems. Integrated glucose-related outcomes have principally been reported in one disease-relevant animal study of parietin, whereas controlled human cardiometabolic intervention studies remain unavailable. Abbreviations: AGE, advanced glycation end product; AMPK, AMP-activated protein kinase; ARE, antioxidant response element; DPP-4, dipeptidyl peptidase-4; eNOS, endothelial nitric oxide synthase; FAS, fatty acid synthase; GLUT4, glucose transporter type 4; HMGCR, 3-hydroxy-3-methylglutaryl-coenzyme A reductase; MAPK, mitogen-activated protein kinase; NF-κB, nuclear factor kappa B; NLRP3, NOD-, LRR- and pyrin domain-containing protein 3; NO, nitric oxide; NRF1, nuclear respiratory factor 1; Nrf2, nuclear factor erythroid 2-related factor 2; PGC-1α, peroxisome proliferator-activated receptor gamma coactivator 1-alpha; ROS, reactive oxygen species; SREBP-1c, sterol regulatory element-binding protein 1c; TFAM, mitochondrial transcription factor A.

A schematic overview of the principal experimental activities reported for lichen-derived compounds, together with their potential relevance to diabetes and cardiovascular risk and the current translational limitations, is provided in Figure 5.

## 5. Lichen-Derived Compounds in Diabetes

The antidiabetic potential of lichen-derived compounds has been explored primarily through studies of carbohydrate-digesting enzymes, protein glycation, oxidative stress, inflammatory signalling, and, less frequently, integrated glucose regulation in vivo. However, the available literature remains dominated by in vitro experiments, molecular docking, and studies of chemically complex extracts. Consequently, these findings are best considered as a basis for pharmacological investigation rather than evidence of established glucose-lowering efficacy [82,83,84,85]. The most directly relevant experimental observations for diabetes and vascular risk are summarised in Table 2.

### 5.1. Glucose Metabolism and Carbohydrate-Digesting Enzymes

Inhibition of intestinal carbohydrate-digesting enzymes is the most directly investigated antidiabetic activity of lichen preparations. In cell-free enzyme assays, purified salazinic acid, norlobaridone, and usnic acid, together with chemically characterised extracts of *Ramalina conduplicans* and *Gondwania regalis*, showed inhibition of α-glucosidase and/or α-amylase [66,68,85,86]. These studies combined enzyme-based screening with different levels of chemical characterisation, while molecular docking was used to explore possible compound–enzyme interactions in the studies of *Hypotrachyna cirrhata*, *R. conduplicans*, and *G. regalis* [66,68,85]. However, differences in tested materials, enzyme sources, assay conditions, extract composition, compound purity, and reporting metrics prevent direct potency comparisons across studies [87,88]. The principal quantitative findings are therefore presented in Table 2. Their translational relevance depends on gastrointestinal stability, achievable luminal exposure, selectivity, tolerability, and confirmation of postprandial glucose lowering in vivo [87].

Among the available studies, Abdella et al. provided one of the few evaluations of an isolated lichen-derived metabolite in a diet-induced model relevant to obesity and glucose dysregulation. Thirty-five male Wistar rats were divided into seven groups of five animals and fed either a standard diet or a high-fat/high-fructose diet for 90 days. The diet-fed groups were left untreated or received parietin at 25, 50, 100, or 200 mg/kg/day or atorvastatin at 10 mg/kg/day by daily gastric gavage. At 100 mg/kg/day, parietin was associated with reductions in intestinal lipase and α-amylase activities, PTP1B activity, body weight, and fasting blood glucose, together with a 112% increase in hepatic glycogen. During the oral glucose tolerance test, blood glucose was approximately 30% and 38% lower than in untreated diet-fed rats at 60 and 90 min, respectively. Parietin was also associated with favourable changes in the reported serum and tissue lipid variables and with reductions in pancreatic inflammatory-enzyme activities, oxidative-stress markers, and immune-cell infiltration. Histological examination suggested reduced lipid accumulation and partial preservation of pancreatic islet morphology, particularly at 100 and 200 mg/kg/day [67]. These findings provide more direct metabolic evidence than chemical, docking, or isolated enzyme-inhibition studies, but several limitations constrain their interpretation. The model was based on prolonged high-fat/high-fructose feeding without direct measurements of circulating insulin or validated indices of insulin sensitivity, and treatment appears to have been administered concomitantly with the obesogenic diet. Therefore, the findings are more appropriately interpreted as preventive effects in a model of diet-induced obesity and glucose intolerance than as treatment of established type 2 diabetes. The small group size, male-only design, absence of a specific antidiabetic comparator, predominantly qualitative histological assessment, and inconsistencies in some reported outcomes further limit the robustness of these findings. Although four parietin doses were evaluated, 100 mg/kg/day was generally identified as the most effective dose without a consistent incremental benefit at 200 mg/kg/day. The relatively high dose associated with the principal metabolic effects further limits direct extrapolation to potential human use. Moreover, compound purity was not quantitatively established, systemic exposure and pharmacokinetic parameters were not measured, and the claim of low toxicity was not supported by a dedicated toxicological assessment. Consequently, the translational relevance and achievable safety margin of the reported effects remain uncertain.

### 5.2. Insulin Signalling and Metabolic Inflammation

Insulin resistance is sustained by defects in insulin-receptor signalling, altered substrate utilisation, ectopic lipid accumulation, and inflammatory stress in liver, skeletal muscle, and adipose tissue [5,89,90]. AMPK, PPARs, IRS–PI3K–Akt signalling, and GLUT4 trafficking are established regulators of metabolic homeostasis and therefore represent relevant pathways for future investigation of lichen-derived metabolites [26,27,90,91,92]. However, the current literature provides little direct evidence that purified lichen compounds improve insulin-stimulated glucose uptake, GLUT4 translocation, hepatic glucose production, or systemic insulin sensitivity. In the diet-induced obesity model examined by Abdella et al., parietin reduced tissue PTP1B activity, an effect interpreted by the investigators as being consistent with improved insulin signalling [67]. Nevertheless, circulating insulin, insulin-receptor phosphorylation, downstream Akt activation, and validated indices of insulin sensitivity were not assessed. The reported improvement in glucose tolerance therefore provides indirect metabolic evidence but does not establish restoration of insulin signalling. Accordingly, these pathways should remain framed as biologically plausible targets rather than established mechanisms of action of parietin or other lichen-derived compounds [83,84].

Anti-inflammatory findings provide an additional connection with insulin resistance. Lobaric acid, (+)-usnic acid, and atraric acid suppressed NF-κB-related signalling, inflammatory mediator production, or NLRP3 inflammasome activation in macrophage and animal inflammatory models [69,71,93]. Because TNF-α, IL-6, IL-1β, and inflammasome signalling can impair insulin action, these observations justify investigation in adipocytes, hepatocytes, myotubes, and obesity-related models. These cell-based inflammatory findings support mechanistic plausibility, but suppression of inflammation in non-metabolic experimental systems cannot be assumed to restore insulin sensitivity in metabolically dysfunctional tissues [5,94,95].

### 5.3. Oxidative Stress and Pancreatic β-Cell Vulnerability

Pancreatic β-cells are susceptible to glucotoxic, lipotoxic, inflammatory, and mitochondrial stress because of their high secretory demand and comparatively limited antioxidant defences [96,97,98]. The radical-scavenging and anti-inflammatory activities reported for selected lichen extracts and isolated metabolites provide a rationale for investigating their effects on β-cell stress responses [69,71,75,76]. Nevertheless, chemical antioxidant capacity cannot be equated with preservation of β-cell function, particularly because usnic acid can perturb mitochondrial function and reduce hepatocyte viability at biologically active concentrations [99,100].

**Table 2:** Selected experimental evidence on lichen-derived materials relevant to diabetes and cardiovascular risk modulation.

Material	Experimental Model and Exposure	Principal Findings	Interpretation and Main Limitations	Refs.
*Ramalina conduplicans* extract	UPLC-Q-ToF-MS/MS profiling; cell-free antioxidant and carbohydrate-digesting-enzyme assays	Free-radical scavenging and inhibition of carbohydrate-digesting enzymes	Chemically characterised extract, but chemical and enzyme endpoints do not demonstrate glycaemic efficacy	[68]
Salazinic acid and norlobaridone	Cell-free α-glucosidase-inhibition assay and molecular docking	α-Glucosidase inhibition and predicted interactions with the enzyme	Purified compounds, but no assessment of intestinal exposure, glucose lowering, or insulin sensitivity in vivo	[66]
Usnic acid isolated from *Usnea* sp.	Cell-free α-glucosidase-inhibition assay	α-Glucosidase inhibition; IC_50_: 106.78 µg/mL (approximately 310 µM)	The required concentration exceeds those associated with mitochondrial perturbation in hepatic experimental systems; in vivo glycaemic efficacy remains untested	[86,99,100]
*Gondwania regalis* ethanolic extract	Metabolomic profiling; antioxidant and enzyme-inhibition assays; molecular docking	Inhibition of α-glucosidase and α-amylase; IC_50_: approximately 2246 and 585 µg/mL, respectively	Chemically profiled extract, but the high inhibitory concentrations and absence of in vivo outcomes indicate screening-level evidence	[85]
Parietin	Male Wistar rats fed a high-fat/high-fructose diet; 25–200 mg/kg/day by gastric gavage for 90 days	At 100 mg/kg/day, hepatic glycogen increased by 112%; OGTT glucose was approximately 30% and 38% lower at 60 and 90 min, respectively. Fasting glucose and several lipid, inflammatory, oxidative, and histological variables also improved	Disease-relevant multi-dose evidence, but groups were small; insulin sensitivity was not directly measured; pharmacokinetics and dedicated toxicity were not assessed	[67]
(+)-Usnic acid	Non-disease-specific animal models of acute and chronic inflammation	Reduction in inflammatory responses	Supports anti-inflammatory activity, but does not demonstrate endothelial protection or cardioprotection; toxicity remains a major concern	[71]
Lobaric acid	Macrophage models of inflammatory signalling and NLRP3 activation	Suppression of NF-κB/MAPK signalling and NLRP3 inflammasome activation	Mechanistically relevant but indirect with respect to endothelial function, vascular injury, or atherosclerotic outcomes	[69]
Selected purified lichen metabolites	Cell-free AGE-formation assays and functional vascular-reactivity experiments	Inhibition of AGE formation and vasodilatory responses	Direct antiglycation and vascular observations, but molecular targets, achievable exposure, selectivity, and safety remain undefined	[82]

Abbreviations: AGE, advanced glycation end product; MAPK, mitogen-activated protein kinase; NF-κB, nuclear factor kappa B; NLRP3, NOD-, LRR- and pyrin domain-containing protein 3; UPLC-Q-ToF-MS/MS, ultra-performance liquid chromatography coupled with quadrupole time-of-flight tandem mass spectrometry.

Direct studies assessing glucose-stimulated insulin secretion, β-cell survival, mitochondrial respiration, or protection from glucolipotoxicity remain scarce. No human study identified in the literature search evaluated glucose-stimulated insulin secretion, β-cell function, or β-cell preservation after administration of a purified lichen-derived compound. Future investigations should therefore combine functional secretory outcomes with intracellular redox and bioenergetic measurements, while defining the concentration range at which any cytoprotective effect remains distinct from nonspecific cellular stress or toxicity.

### 5.4. Current Evidence and Knowledge Gaps

Taken together, the available evidence supports a broad but still preliminary antidiabetic profile. The most direct experimental findings concern inhibition of carbohydrate-digesting enzymes and AGE formation, whereas integrated glucose-related outcomes have been investigated principally in the parietin diet-induced model [66,67,82]. Direct effects on insulin sensitivity and β-cell secretory function remain insufficiently investigated. Comparisons across studies are further complicated by differences in lichen species, extraction procedures, chemical characterisation, enzyme sources, assay conditions, exposure concentrations, and outcome reporting [68,85,87,88].

Disease-relevant animal evidence remains limited, with the parietin investigation representing the principal recent study of a purified lichen-derived metabolite reporting integrated glucose-related outcomes [67]. No controlled human intervention study of a purified lichen-derived compound in insulin resistance, prediabetes, or type 2 diabetes was identified in the literature search. Further development will require chemically standardised preparations or purified compounds, pharmacokinetic characterisation, demonstration of metabolic effects at achievable exposures, and simultaneous evaluation of hepatic and mitochondrial safety, particularly for usnic-acid-containing preparations [18,19].

## 6. Lichen-Derived Compounds in Cardiovascular Risk Modulation

The potential cardiovascular relevance of lichen-derived compounds has been considered mainly in relation to endothelial dysfunction, oxidative stress, inflammation, and lipid-related vascular injury. These processes are central to the development of atherosclerotic cardiovascular disease, particularly in the presence of insulin resistance and diabetes [4,8,101]. However, most studies of lichen metabolites were not designed in cardiovascular disease models. Their vascular implications should therefore be discussed as an emerging area of investigation rather than as evidence of established cardiovascular protection [58,84]. The principal experimental observations relevant to vascular function are presented alongside the diabetes-related evidence in Table 2.

### 6.1. Endothelial Function and Vascular Reactivity

Endothelial dysfunction links metabolic abnormalities to vascular disease through reduced nitric oxide (NO) bioavailability, increased oxidative stress, inflammatory activation, and impaired regulation of vascular tone [8,102]. Experimental antioxidant and anti-inflammatory findings provide a rationale for investigating the endothelial effects of lichen-derived compounds. Selected purified metabolites have shown antioxidant capacity in vitro, although compound- and cell-dependent cytotoxicity was also observed [103]. These findings do not demonstrate preservation of endothelial function because they were obtained outside endothelial or disease-specific vascular systems.

More direct vascular observations are available from a study in which a panel of natural and synthetically modified lichen metabolites was evaluated not only for inhibition of advanced glycation end-product formation but also for vasodilatory activity. Selected compounds produced vasodilatory responses in experimental vascular-reactivity assays, providing preliminary functional evidence of an effect on vascular tone under the conditions employed [82]. Nevertheless, these findings remain insufficient to establish endothelial protection, because the responsible targets, achievable vascular exposure, selectivity, and safety margins require further characterisation. Studies in endothelial cells and intact vessels should assess eNOS activation, NO production, oxidative balance, leukocyte adhesion, and concentration-dependent vasoreactivity while simultaneously excluding cytotoxic or mitochondrial effects [8,19,102].

### 6.2. Oxidative, Inflammatory, and Atherosclerotic Processes

Oxidative modification of lipoproteins, endothelial activation, monocyte recruitment, and macrophage inflammatory responses contribute to atherosclerotic plaque formation and progression [4,101,104]. Selected lichen extracts and isolated metabolites showed radical-scavenging or lipid-peroxidation-inhibitory activity in chemical and cellular assays [75,76,85]. In separate inflammatory models, lobaric acid and atraric acid suppressed NF-κB-related signalling, inflammatory-mediator production, or NLRP3 inflammasome activation [69,93]. These chemical and cell-based observations support potential relevance to vascular injury, but most were obtained outside disease-specific models of atherosclerosis and therefore do not demonstrate an effect on plaque development or progression.

Direct evidence concerning LDL oxidation, endothelial adhesion molecules, foam-cell formation, macrophage cholesterol handling, smooth-muscle-cell responses, or plaque characteristics remains sparse; reductions in ROS or inflammatory mediators should therefore not be interpreted as evidence of an effect on atherosclerotic plaque development [58,84]. The same caution applies to inflammasome inhibition: although NLRP3 contributes to atherosclerotic inflammation, activity in macrophage or acute-inflammation models does not establish modulation of the arterial wall [4,30,105]. Disease-relevant studies should incorporate modified-lipoprotein uptake, macrophage phenotype, endothelial–leukocyte interactions, and plaque-level outcomes rather than relying exclusively on antioxidant or cytokine assays [106,107].

### 6.3. Lipid Metabolism and Cardiovascular Risk

Evidence that lichen-derived compounds directly modify circulating lipids or lipoprotein metabolism is particularly limited. AMPK and PPAR signalling regulate fatty-acid oxidation, hepatic lipid synthesis, adipose-tissue function, and triglyceride turnover, but direct engagement of these pathways by purified lichen metabolites has not been established in appropriate metabolic models [108,109,110]. The ability of selected lichen extracts or metabolites to limit lipid peroxidation under experimental assay conditions is distinct from reducing circulating LDL cholesterol, triglycerides, or apolipoprotein B-containing particles [75,76,111].

Available studies do not provide a consistent basis for concluding that lichen preparations improve lipoprotein production, clearance, remodelling, or reverse cholesterol transport [84]. Future work should quantify conventional lipid variables together with apolipoproteins, lipoprotein particle characteristics, hepatic lipid handling, and systemic exposure to the tested metabolite. Until such data are available, lipid-modulating effects should remain a research hypothesis rather than a defining pharmacological property of lichen-derived compounds.

### 6.4. Current Knowledge and Research Needs

Overall, the cardiovascular literature is less developed than the chemical, antioxidant, and anti-inflammatory literature surrounding lichens. Preliminary vasodilatory observations and the capacity of selected compounds to modulate redox and inflammatory responses justify further vascular investigation, but direct evidence regarding endothelial function, atherosclerotic plaque development, or lipid-related cardiovascular risk remains limited [58,82,84]. No human intervention study identified in the literature search evaluated the effects of a purified lichen-derived compound on vascular biomarkers, endothelial function, arterial disease, or cardiovascular outcomes.

Progress in this field will require chemically defined preparations, exposure measurements, vascular models with functional endpoints, and integrated assessment of hepatic and mitochondrial safety. This is especially important for usnic acid, for which mitochondrial uncoupling, ATP depletion, and loss of hepatocyte viability have been demonstrated experimentally [99,100]. At present, lichen metabolites are more appropriately viewed as candidate vascular pharmacological scaffolds than as interventions for cardiovascular prevention or treatment.

## 7. Translational Challenges and Future Development

### 7.1. Bioavailability, Pharmacokinetics, and Formulation

The main translational challenge is to determine whether pharmacologically relevant concentrations can reach target tissues without unacceptable systemic exposure. Usnic acid illustrates this problem particularly clearly because its limited aqueous solubility, marked lipophilicity, stereochemical complexity, and mitochondrial toxicity complicate systemic development [17,19,112,113]. In LPS-stimulated RAW264.7 macrophages, inhibition of TNF-α accumulation and nitric oxide production was reported at IC_50_ values of 4.7 and 12.8 µM, respectively [114]. By contrast, uncoupling of oxidative phosphorylation was observed in isolated rat liver mitochondria at concentrations of 0.15–6 µM [99], while exposure of primary rat hepatocytes to 10–30 µM reduced cellular ATP, with a marked loss of viability at 30 µM after 24 h [100]. Moreover, the reported α-glucosidase IC_50_ of 106.78 µg/mL, corresponding to approximately 310 µM, is substantially higher than concentrations associated with mitochondrial perturbation in hepatic experimental systems [86]. Although these findings were obtained in different experimental models and cannot be used to calculate a formal therapeutic index, their overlap provides no evidence of a clearly separated efficacy-toxicity window for systemic cardiometabolic use.

Available pharmacokinetic data are exclusively preclinical and do not resolve this uncertainty. In rabbits, intravenous administration of 5 mg/kg produced a terminal plasma half-life of 10.7 ± 4.6 h, whereas after oral administration of 20 mg/kg the terminal half-life was 18.9 ± 2.9 h and the estimated absolute bioavailability was approximately 77.8% [115]. In rats, the absolute oral bioavailability of purified usnic acid was reported to be 69.2%, whereas administration within an ethanolic Usnea longissima extract produced a reported apparent bioavailability of 146.9%, suggesting a substantial matrix-dependent alteration of systemic exposure [116]. In vitro, usnic acid is extensively protein-bound, exceeding 99% in human and rat plasma, and inhibits several drug-metabolising enzymes, particularly CYP2C9 and CYP2C19 [117]. Metabolic studies have identified hydroxylation, methylation, glucuronidation, and the formation of potentially reactive metabolites, with CYP2C8, CYP2C9, CYP3A4, and UGT1A1 contributing to its biotransformation [118,119]. However, no human pharmacokinetic study has defined systemic or target-tissue exposure, clearance, accumulation during repeated administration, or exposure associated with either cardiometabolic activity or toxicity. Comparable pharmacokinetic information is much more limited for depsides, depsidones, and other lichen metabolites [14].

Exposure and biological activity may vary with species identity, extraction procedure, matrix composition, purity, vehicle, formulation, and batch composition. Chemical characterisation and quantitative profiling, including LC–MS/MS-based approaches, are therefore essential for linking composition to reproducible biological effects [49,68,120]. Formulation strategies and synthetic derivatives should be evaluated simultaneously for solubility, systemic and tissue exposure, metabolic stability, efficacy, and hepatic safety; increased in vitro potency alone does not demonstrate translational progress [19,121]. Recent work with other natural bioactives illustrates the potential of targeted formulation strategies: mesenchymal stem-cell-membrane-coated kaempferol nanoparticles showed preferential targeting of inflamed endothelial environments and improved atherosclerotic outcomes in an experimental model, suggesting that biomimetic delivery may represent a strategy worth investigating for poorly bioavailable lichen metabolites [122]. Such approaches would nevertheless require evaluation of nano–bio interfacial behaviour, because surface properties and protein-corona composition can influence endothelial uptake and vascular distribution under haemodynamic conditions [123]. Indeed, formulation-mediated increases in bioavailability could also increase hepatic exposure and toxicity unless a corresponding improvement in the therapeutic window is demonstrated.

### 7.2. Safety, Toxicity, and Drug Interactions

Safety remains the most immediate obstacle to clinical development of usnic acid. Experimental studies using purified usnic acid support a direct toxicological mechanism involving mitochondrial uncoupling, ATP depletion, oxidative stress, and loss of hepatocyte viability [99,100]. Human evidence, however, largely consists of case reports and case series involving weight-loss supplements rather than controlled exposure to purified usnic acid [18,124]. Seven previously healthy individuals developed acute hepatotoxicity within three months of using LipoKinetix [125], and additional reports described submassive hepatic necrosis or fulminant hepatic failure requiring liver transplantation [126]. LipoKinetix contained sodium usniate together with norephedrine, yohimbine, 3,5-diiodothyronine, and caffeine; consequently, these cases establish a serious safety signal for usnic-acid-containing products but do not permit unequivocal attribution of the clinical injury to purified usnic acid alone. Formulation effects, co-exposures, dose, duration, enantiomeric form, and individual susceptibility must therefore be considered when interpreting the human evidence. More broadly, the experience with herbal and dietary supplement-associated liver injury reinforces the need for analytical standardisation, prospective monitoring, and structured causality assessment [127,128].

Cytotoxicity reported for lichen metabolites in cancer-cell models cannot be extrapolated directly to metabolic or vascular tissues, but it indicates that efficacy, cellular selectivity, and safety margins must be examined together [129,130]. This is particularly relevant to people with diabetes or elevated cardiovascular risk, in whom steatotic liver disease, kidney impairment, and polypharmacy may increase the consequences of hepatic or mitochondrial toxicity [1,3]. Accordingly, toxicological evaluation should use metabolically and vascularly relevant cell systems, repeated-dose animal studies, and parallel measurements of efficacy, systemic exposure, mitochondrial function, and organ injury.

Evidence regarding interactions with cardiometabolic therapies is also limited. Purified usnic acid inhibited CYP2C9 and CYP2C19 in vitro [117], but the clinical relevance of this finding has not been established. Direct interaction studies with antidiabetic, lipid-lowering, antihypertensive, or antithrombotic drugs are lacking, while information concerning transporters and concentration-dependent protein-binding displacement remains insufficient [19]. Before co-administration is considered, compound-specific studies should address absorption, distribution, metabolism, excretion, enzyme inhibition or induction, transporter effects, and toxicity during co-exposure. This is clinically relevant because patients with cardiometabolic disease commonly receive several concurrent therapies [131,132].

Taken together, current evidence does not establish a biologically achievable exposure window separating systemic pharmacological activity from mitochondrial and hepatic toxicity. Demonstration of such a window, based on unbound drug concentrations, repeated-dose pharmacokinetics, target-tissue exposure, and parallel efficacy and toxicity endpoints, should be considered a prerequisite for further systemic development of usnic acid in diabetes or cardiovascular risk modulation.

### 7.3. Standardisation and Experimental Development

Future development requires taxonomically authenticated, chemically defined, and reproducible test materials. Lichen identity should be established by a qualified specialist using accepted macromorphological, anatomical, and microscopic criteria, supported where appropriate by chemical characters obtained through spot tests, thin-layer chromatography, or analytical profiling [133]. A voucher specimen should be deposited in a recognised herbarium or institutional collection, with the accession number, collector, collection date, geographical origin, substrate, and relevant environmental information reported [133,134]. For fragmentary material, morphologically ambiguous specimens, or taxa belonging to closely related species complexes, sequencing of the fungal internal transcribed spacer region may complement morphological and chemical identification, provided that sequences are compared with curated, voucher-supported reference data [133,134]. Molecular barcoding should be regarded as complementary rather than automatically definitive because discrimination between closely related lichen-forming fungi may remain incomplete and identification success depends strongly on the taxonomic and geographical coverage of the reference database [133,134]. Authentication should refer to the same biological batch used for extraction, thereby allowing taxonomic identity, chemical composition, and biological activity to be traced to the same source material [68,120]. Studies should additionally report extraction procedures, extraction yield, quantitative chemical fingerprints, major constituent concentrations, stability, batch-to-batch variability, and contaminant control [49,68,120]. Integrated metabolomic and network-pharmacology approaches may help prioritise candidate metabolites within complex extracts, but computational binding predictions and pathway associations require experimental validation in disease-relevant systems [14,66,68,83].

Initial experiments should progress beyond cell-free antioxidant assays and molecular docking to concentration–response studies in adipocytes, hepatocytes, myotubes, pancreatic β-cells, macrophages, and endothelial cells, with explicit cytotoxicity thresholds. Proposed effects on AMPK, NF-κB, Nrf2, PI3K–Akt, eNOS/NO, and lipid metabolism should be tested causally through pathway inhibitors, genetic perturbation, or validated target-engagement biomarkers. Animal studies should incorporate positive pharmacological controls, systemic exposure measurements, liver and kidney safety, and functional metabolic and vascular endpoints. Pleiotropic activity may be valuable, but it must be distinguished from nonspecific cellular stress or toxicity [19,59].

### 7.4. Clinical Translation and Research Priorities

Only compounds that show reproducible target engagement and efficacy at achievable, non-toxic exposure should progress toward clinical investigation. If supported by a convincing preclinical package, early human studies should prioritise safety, tolerability, pharmacokinetics, interaction potential, and prespecified biomarkers. Participants with stable prediabetes or metabolic syndrome may eventually provide informative study populations, but only after hepatic safety and co-administration risks have been adequately addressed [135].

Medicinal-chemistry studies illustrate that the usnic acid scaffold can be structurally modified, although the resulting evidence remains largely outside the cardiometabolic field. Bazin et al. synthesised nine usnic acid–amine conjugates and found that several polyamine derivatives displayed cytotoxic activity in tumour-cell models, with the 1,8-diaminooctane derivative showing the greatest activity and inducing apoptosis [121]. In a different experimental context, Shi et al. developed a series of usnic acid derivatives targeting tau aggregation and neuroinflammation. Their lead compound inhibited tau aggregation, reduced nitric oxide release in LPS-stimulated BV2 microglial cells relative to sodium usnate, and showed protective activity in a rat model of okadaic acid-induced memory impairment [136]. These studies demonstrate that derivatisation can alter the biological activity of the usnic acid scaffold, but neither investigated glucose regulation, vascular function, pharmacokinetics, mitochondrial uncoupling, or hepatic safety. Future optimisation should therefore evaluate solubility, metabolic stability, tissue exposure, target selectivity, and toxicity alongside diabetes- and cardiovascular-relevant functional endpoints. Combination strategies and precision-nutraceutical applications remain speculative because exposure, interaction, standardisation, and co-administration safety data are lacking [19,132]. At present, lichen metabolites are more appropriately regarded as pharmacological probes and chemical scaffolds than as interventions ready for clinical use [13,137].

Practical and commercial applications of lichen-derived materials already exist, although they remain largely unrelated to cardiometabolic medicine. Preparations of *Cetraria islandica* (Iceland moss), including comminuted thallus and aqueous or ethanolic extracts, are used in European traditional herbal medicinal products as demulcents for oral and pharyngeal irritation with associated dry cough and for temporary loss of appetite. These indications are recognised on the basis of longstanding traditional use rather than robust evidence from controlled clinical trials [138]. Extracts of *Evernia prunastri* (oakmoss) and *Pseudevernia furfuracea* (treemoss) also have established commercial applications as fragrance materials. However, their use is subject to safety restrictions because atranol and chloroatranol, naturally occurring constituents associated with contact sensitisation, are prohibited in cosmetic products in the European Union [139]. These examples confirm that lichen-derived preparations have progressed beyond laboratory investigation in selected sectors, but they do not provide evidence of efficacy, systemic exposure, or safety for diabetes or cardiovascular-risk reduction. No authorised cardiometabolic medicine based on a lichen extract or purified lichen metabolite was identified in the present literature search.

## 8. Conclusions

Lichens provide a chemically distinctive source of secondary metabolites with activities that intersect several mechanisms relevant to diabetes and cardiovascular risk. Depsides, depsidones, dibenzofurans, anthraquinones, and related phenolic compounds have shown antioxidant, anti-inflammatory, enzyme-inhibitory, antiglycation, and vasomodulatory effects in experimental systems. These findings support their continued investigation as pharmacological probes and medicinal-chemistry scaffolds. However, the available evidence remains predominantly preclinical and uneven across compounds and disease domains. Chemical radical-scavenging, molecular docking, or inhibition of digestive enzymes can identify promising candidates but does not establish improved insulin sensitivity, β-cell preservation, endothelial function, atherosclerosis, or cardiovascular outcomes. Several proposed mechanisms, including modulation of AMPK, PPARs, Nrf2, NF-κB, NLRP3, and eNOS, remain incompletely validated in diabetes- or cardiovascular-relevant models. Human efficacy data are essentially absent.

Translation will require reproducible chemical standardisation, pharmacokinetic characterisation, demonstration of activity at achievable non-toxic exposure, and rigorous evaluation of hepatic and mitochondrial safety. On the basis of the currently available evidence, parietin and lobaric acid emerge as the most appropriate compounds for focused preclinical investigation, although for different reasons. Parietin currently provides the most direct disease-relevant evidence because it improved integrated glucose- and lipid-related outcomes in a diet-induced animal model. Its development is nevertheless limited by the high effective dose, uncertain purity, absence of pharmacokinetic data, and lack of a dedicated toxicological assessment. Lobaric acid provides comparatively well-defined compound-specific evidence of NF-κB/MAPK and NLRP3 modulation, but its potential metabolic and vascular effects remain to be tested in disease-relevant models. Usnic acid warrants a distinct priority as a pharmacologically characterised scaffold and as a benchmark for studying the relationship between activity, exposure, and toxicity. However, its mitochondrial and hepatic safety liabilities argue against prioritising the unmodified compound for systemic cardiometabolic administration; further investigation should instead focus on safer derivatives or targeted formulations designed to improve the therapeutic window. These priorities reflect the relative maturity and specific limitations of the available evidence rather than readiness for clinical translation. At present, no lichen-derived compound can be recommended for the prevention or treatment of diabetes or for cardiovascular risk reduction.

## Data Availability

No new data were created or analyzed in this study. Data sharing is not applicable to this article.
